# Application of Protein-Based Films and Coatings for Food Packaging: A Review

**DOI:** 10.3390/polym11122039

**Published:** 2019-12-09

**Authors:** Hongbo Chen, Jingjing Wang, Yaohua Cheng, Chuansheng Wang, Haichao Liu, Huiguang Bian, Yiren Pan, Jingyao Sun, Wenwen Han

**Affiliations:** 1 College of Electromechanical Engineering, Qingdao University of Science and Technology, Qingdao 266061, China; chenhb@qust.edu.cn (H.C.); 4108030063@mails.qust.edu.cn (J.W.); qustcyh2013@126.com (Y.C.); wangcs07@163.com (C.W.); bhg@qust.edu.cn (H.B.); pyr90hot@163.com (Y.P.); 2 Shandong Provincial Key Laboratory of Polymer Material Advanced Manufactorings Technology, Qingdao University of Science and Technology, Qingdao 266061, China; 3 Academic Division of Engineering, Qingdao University of Science & Technology, Qingdao 266061, China; liuhaichao66mm@qust.edu.cn (H.L.);; 4 College of Mechanical and Electrical Engineering, Beijing University of Chemical Technology, Beijing 100029, China; 5 National Engineering Laboratory for Advanced Tire Equipment and Key Materials, Qingdao University of Science and Technology, Qingdao 266061, China

**Keywords:** protein-based films and coatings, properties, plasticizers, applications for food-packaging, active protein-based films

## Abstract

As the IV generation of packaging, biopolymers, with the advantages of biodegradability, process ability, combination possibilities and no pollution to food, have become the leading food packaging materials. Biopolymers can be directly extracted from biomass, synthesized from bioderived monomers and produced directly by microorganisms which are all abundant and renewable. The raw materials used to produce biopolymers are low-cost, some even coming from agrion dustrial waste. This review summarized the advances in protein-based films and coatings for food packaging. The materials studied to develop protein-based packaging films and coatings can be divided into two classes: plant proteins and animal proteins. Parts of proteins are referred in this review, including plant proteins i.e., gluten, soy proteins and zein, and animal proteins i.e., casein, whey and gelatin. Films and coatings based on these proteins have excellent gas barrier properties and satisfactory mechanical properties. However, the hydrophilicity of proteins makes the protein-based films present poor water barrier characteristics. The application of plasticizers and the corresponding post-treatments can make the properties of the protein-based films and coatings improved. The addition of active compounds into protein-based films can effectively inhibit or delay the growth of microorganisms and the oxidation of lipids. The review also summarized the research about the storage requirements of various foods that can provide corresponding guidance for the preparation of food packaging materials. Numerous application examples of protein-based films and coatings in food packaging also confirm their important role in food packaging materials.

## 1. Introduction

In order to prolong the shelf life of food and ensure the quality of this food during transportation, food packaging has become increasingly important. The synthetic films occupy a large proportion in the field of food packaging for their good gas and liquid barrier characteristics and mechanical properties, low-cost and durability. However, the resistance to degradation of synthetic films made of petroleum results in serious environmental pollution, called white pollution [1]. 

Thus, the large consumption of raw materials increases the consumption of petroleum resources and disposal problems [2,3]. The use of incineration to deal with the used synthetic films will also generate a lot of heat and toxic gases, which poses a serious threat to human survival and health. In addition, the non-renewable nature of petroleum may make the price of raw materials rise in the future [4]. Recently, 15 countries and regions (including New Zealand, Korea, New York, Chile, France, European Union member states, Australia, India, Britain, Macao, Iceland, Washington State, Brazil and Hainan) have issued bans on plastic products, aiming to curb the increasingly serious marine and ecological and environmental pollution caused by waste plastic products. Most of these countries mention the ban on single-use plastic bags, which are made from nondegradable synthetic films.

All these have accelerated the development of biodegradable food packaging materials made of cheap and readily available raw materials. As the IV generation of packaging, biopolymers, with the advantages of biodegradability, processability, combination possibilities and no pollution to food, have become the leading food packaging materials. Some biopolymers would even act as compost and soil conditioners [5].

**Figure 1:**
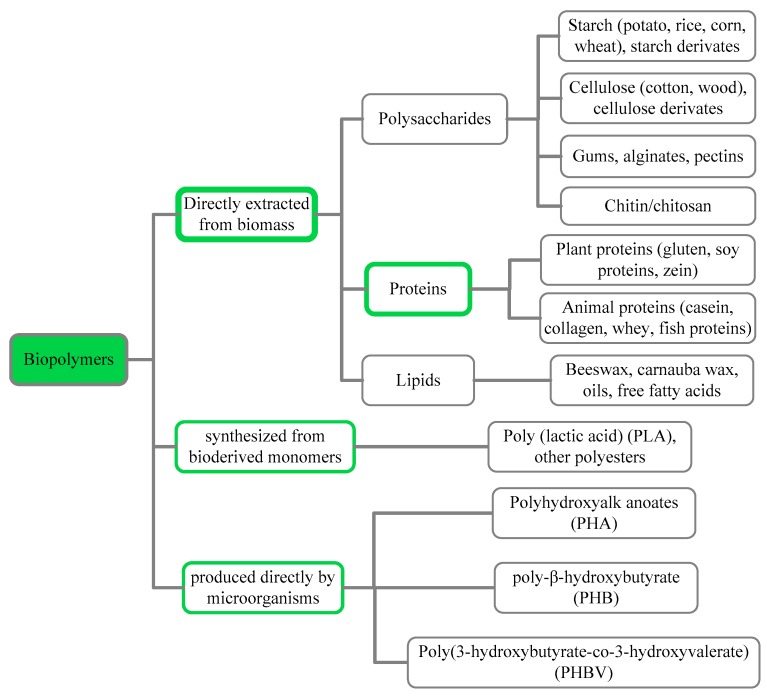
Categories and origins of the biopolymers.

As shown in Figure 1, there are three main ways to get biopolymers: (1) Biopolymers (polysaccharides, proteins and lipids) can be directly removed/extracted from biomass;(2) biopolymers (polylactide (PLA) and other polyesters) can be obtained through synthesizing from bioderived monomers; and (3) biopolymers (polyhydroxyalkanoate (PHA), polyhydroxybutyrate (PHB) and poly (3-hydroxybutyrate–*co*–3-hydroxyvalerate) (PHBV) can be produced directly by microorganisms those are all abundant and renewable [6]. The raw materials used to produce biopolymers are low-cost, and some even come from agrion dustrial waste.

**Figure 2:**
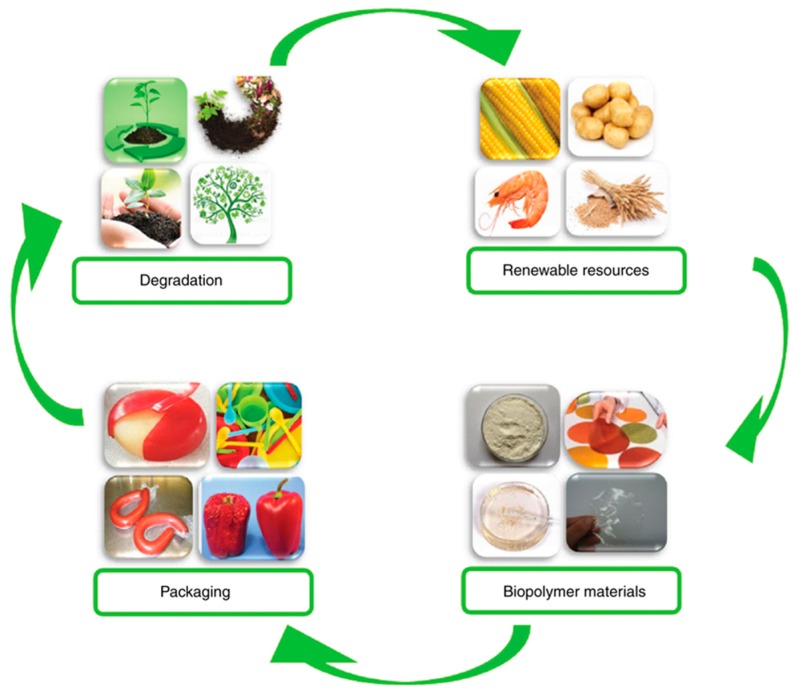
Life Cycle of Biopolymer Packaging Materials. Reproduced with permission from [6].

Biopolymers are not only degradable, but the raw materials are renewable resources. Biopolymers come from nature and eventually return to nature. As shown in Figure 2, the biopolymers used in our life can form a closed cycle, one cycle complete and then go on to the next.

Biopolymers used as food packaging materials that include films and coatings (films are applied onto the food surface after prepared separately, whereas coatings are formed directly on the food surface) must ensure the health safety, mechanical, thermal and barrier properties and durability. Therefore, in addition to biodegradability, the functionality of the biopolymers-based material is also important [7]. The complex chemical structure and side chains structure provide opportunities for the improvement of biopolymer-based materials through various physical and chemical means, in order to meet the needs in particular situations [8].

## 2. Proteins for Biodegradable Films

The materials used in the preparation of biopolymers are based upon polysaccharides, proteins, or lipids. The main advantages of polysaccharides are abundance, availability, low cost, nontoxicity and thermo-processability [9]. However, because of the hydrophilicity of polysaccharides, polysaccharide-based films have poor barrier properties to water vapor [10]. The biggest advantage of lipids is their hydrophobicity, which can improve the barrier property of materials to water vapor. Lipid films are generally used as coatings because lipid-based films are relatively inelastic [11]. There are some advantages of proteins, such as relative abundance, good film-forming ability, high nutritional value, and so on, which make proteins be used extensively for preparing biodegradable films [12]. Compared to polysaccharides and lipids, protein-based polymers are the most useful, because of the excellent gas barrier properties. The oxygen permeability of soy protein-based films is 260, 500, 540 and 670 times lower than that of low-density methyl cellulose, polyethylene, starch and pectin, respectively. Besides, the mechanical properties of protein-based films are also better than those of polysaccharide-based and lipid-based films [13].

Each protein contains about 20 amino acid monomers. Commonly, proteins consist of hundreds of amino acids. The multilevel structures of proteins result in interactions and adhesions between different types of amino acids with different energies at various positions [14]. These structures of proteins can be improved by a variety of physical and chemical methods, for example, mechanical treatment, heat, irradiation, pressure, lipid interfaces, metal ions, acids and alkalis agents [4].

Besides, due to the intrinsic properties of the proteins, they are excellent materials for the preparation of edible films [15,16,17,18,19,20]. Covering foods with edible films can effectively prevent the loss of moisture and flavors, control gas (oxygen, carbonic dioxide and ethylene) exchange and transport active substances (e.g. antimicrobials, antioxidants, or nutraceuticals) [21].Moreover, it is important that nitrogen sources for those that can act as fertilizer can be provided during the degradation of protein-based films, which is an advantage that non protein-based films do not have [22].

The excellent gas barrier qualities and satisfactory mechanical properties make the protein-based biopolymers one of the most potential materials for food packaging. However, the materials possess poor barrier characteristics for water. Addition of some other biomaterials can reduce the sensitivity of the protein-based biopolymer materials to moisture [23]. Because the cohesive energy density of proteins is strong, the protein-based biopolymers are brittle [24]. The fragility and brittleness exhibited during thermo formation make the protein-based films present poor mechanical properties regarding their processability and end-use application [25]. However, natural and/or biodegradable plasticizers possessing good compatibility with protein-based biopolymers could improve the viscoelasticity and extensibility, so as to solve the above problem [26].

The plasticizer was defined by the council of the International Union of Pure and Applied Chemistry (IUPAC) as “a substance or material incorporated in a material (usually a plastic or elastomer) to increase its flexibility, workability, or distensibility’’ [27]. In order to form good films, the plasticizers which are low. Volatile small molecules are highly essential to add during the preparation of raw material. The addition of plasticizers into polymeric materials can make the three-dimensional structure modified, the attractive intermolecular forces decrease and the chain mobility and free volumes increase [28]. Plasticizers can enter between the polymeric molecular chains, associating with polymers through physical and chemical reactions, making the extensibility, flexibility, elasticity and distensibility increase, and also causing the mechanical properties, cohesion and rigidity to decrease [29].

The unique structure (based on 20 different monomers) confers a wider range of functional properties to proteins, especially a high intermolecular binding potential [30]. Protein-based films and coatings can form bonds at different positions and offer great potential to form numerous linkages [31]. The molecular weight, as well as the number and positions of the hydroxyl groups of plasticizers, are all variables that affect their plasticizing ability to protein-based polymers [13]. Therefore, the plasticizers that different types of protein-based biopolymers apply are different, and the effects of the same plasticizer on different protein-based biopolymers are also different.

In general, because of the hygroscopicity of biopolymers and plasticizers, the moisture content of films and coatings is affected by the ambient conditions. In addition, as the main solvent of natural biopolymer technology, water makes the *T*_g_ reduced, and the free volume of biopolymers is increased. Therefore, water is considered as the most powerful plasticizer for hydrocolloid-based films and coatings [32].

Besides water, polyols, monosaccharides, disaccharides and oligosaccharides are the most commonly used plasticizers. Among them, polyols have been reported to be particularly effective for the plasticized hydrophilic polymers [33]. Glycerol (GLY), a typical polyol, has a high hygroscopicity, which can be added to the film-forming solution to improve the brittleness of the films [34]. There are many other materials that can be used as plasticizers for biodegradable films and coatings, such as polyols, including ethylene glycol (EG), tri ethylene glycol (TEG), diethylene glycol (DEG), polyethylene glycol (PEG)and tetra ethylene glycol [30,35,36,37,38,39,40], along with propylene glycol (PG) [38], xylitol [33,41], sorbitol [30,33,34,35,38] and mannitol [42]; monosaccharides, including fructose, mannose, glucose and sucrose [30,33,43,44]; fatty acids [45,46,47]; ethanolamine (EA) [48]; triethanolamine (TEA) [37]; urea [49]; amino acids [50], and so on.

Although there are so many types of plasticizers the most suitable and commonly used plasticizers for different protein-based films are not the same, and the plasticizer selected for the same protein films is also different in different applications. The selection of plasticizers for some kinds of protein-based films in certain applications is mentioned in Table 1.

Although the properties of plasticized protein-based films have been improved, there is still a gap between them and synthetic polymer films in terms of functional properties (as shown in Table 2). On the balance of environmental pressure and functional properties, biodegradable materials are more promising, and are gradually replacing the synthetic polymers. At present, in some food packaging applications, biodegradable protein-based materials have met their requirements. Additionally, with the development of research on biodegradable materials, there will be promising potential measures further improving the various properties of protein-based films. The most appropriate protein source, additives and methods will be chosen according to the nature and requirements of the food, the nature and degree of the protection required, the shelf life, the environmental impact, and so on.

### 2.1. Gluten

Based on the difference in solubility in aqueous alcohols, gluten proteins are mainly divided into two groups: gliadin and glutenin. The former is soluble and the latter is insoluble [62]. The good film-forming property of gluten is based on its cohesiveness and elasticity.

Gluten films are usually obtained by two methods: One is casting that is done in a thin layer, and the drying process is carried out with aqueous alcoholic after casting, and the other is boiling the protein solutions, and then collecting the films formed on the surface of the solutions or thermos pressing [63,64,65]. In general, the characteristics of cast films and thermos pressed films are different: the former has higher elongation properties and the latter has stronger rupture resistance. The stress-strain relationship of films obtained through the two methods is also different, indicating that the network structure of proteins is affected by the production process [66].

The most commonly used solvent in film forming solution is water ethanol. The uniformity of the films can be controlled by adjusting the alkaline or acidic conditions of the film-forming solutions. The tensile strength of films produced from alkaline solutions is significantly higher than that of films produced from acidic solutions [67]. In contrast, the properties of films made from alkaline solutions are worse than that of the films produced through ethanol solution [17]. In addition, heating and mechanical mixing also help disperse the gluten and improve the characteristics of the films [68].

**Figure 3:**
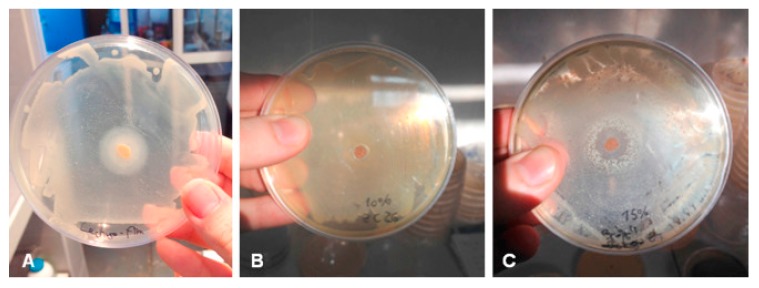
Antimicrobial property of (**a**) control film with lettuce microflora; (**b**) 10% thyme essential oil (TO) film with *Escherichia coli* and (**c**) 15% TO film with broccoli microflora. Reproduced with permission from [71].

The surface of gluten films is shiny, and the films have good oxygen isolation performance, a limited resistance to water vapor and limited mechanical properties [69]. While this is true, various measures can be taken to improve the barrier and mechanical properties of the gluten-based films. The addition of the nonpolar hydrophobic substance (such as mineral oil) into the film-forming dispersion can reduce the water vapor permeability by 25% compared to the control group [70]. Thermal treatment for the casting films that were obtained through covalent crosslinking of gliadin polypeptide chains could also improve the mechanical properties of the gluten-based films [59]. The gluten-based films can also act as active layers in the form of edible films. Thyme essential oil (TO) was added to gluten-based edible films to improve in vitro antioxidant and antimicrobial properties of concentrated samples (as shown in Figure 3) [71]. It can be seen from the figure that with the increase of concentration of TO, the inhibitory effect is enhanced; there is a clear inhibition zone when the concentration of TO is greater than or equal to 10 wt%; the higher concentration of TO in films, the more bacteriostatic species and the larger inhibition zone.

### 2.2. Soy Proteins

Their excellent film-forming property made soy proteins extensively studied. Soy protein films have many functional characteristics, such as adhesiveness, cohesiveness, dough, emulsification, water and fat absorption, fiber formation and texturizing capability [72]. Most of the soy protein films are based on soy protein isolate (SPI), that is a highly refined soy protein containing 90% protein at least, and is made from soy flour, removing the great mass of non-protein components, carbohydrates and fats through isoelectric precipitation [73]. Soy protein films also can be made from soy flour, soymilk and fractioned proteins [74,75]. The soy protein films produced from different soy protein fractions exhibit different properties. The increase of the molecular weight of soy proteins made the tensile strength and elongation increase, but did not make the water vapor barrier properties change [75].

Several methods for the film-forming of soy protein were reported, including heating, extruding, spinning, casting and thermally compacting. Heating had been utilized to form soy protein–lipid films in ancient China [72]. A creamy yellow film formed after the soy milk heating to near boiling was removed and dried, and there finally formed the soy protein film [76]. SPI, polyethylene oxide (PEO) and low-density polyethylene (LDPE) could be extruded to produce soy protein films [77]. Spinning was carried out based on an alkaline dope mixed water, SPI and sodium hydroxide solution. The dope was extruded through a spinning nozzle and poured into acetate buffer (pH 4.7), the proteins in the dope coagulated, and a wet film was formed [78]. Mixtures of glycerol and SPI could be formed into soy protein films through thermally compacting at 150–160 °C and 10 MPa [79]. However, the most commonly used method for preparing soy protein films is casting, that is, drying the thin layers of soy proteins based on film-forming solutions [4]. 

The film-forming solutions of soy protein films are acid or alkaline, but the characteristics of films produced by alkaline solutions is better than that of films produced by acid solutions [2,80]. In addition, different protein concentrations of film forming solutions are required for different film-forming methods. Commonly, the protein concentration is required 4–5% and 80% for wet processing and dry processing, respectively [81].

The addition of some substances can affect the film-formation abilities of soy proteins. For example, the addition of sodium dodecyl sulfate (SDS) makes the extendibility of the films substantially increase, while simultaneously the moisture barrier properties improved [28,82]; the addition of carboxy methyl cellulose (CMC) improves the water vapor permeability significantly [83,84]; the addition of cysteine makes the number of disulfide bonds of the film-forming solutions and the tensile strength of films increase.

In addition, post-treatments after film formation, such as irradiation and heat curing, can also improve the performance of the soy protein films. The bityrosine can be formed between two protein chains through **γ**-irradiation to make the mechanical properties of soy protein films increase. The formation of hydrophobic and disulfide bonds through heat treatment can also make cross-linking promoted [83].

Heat curing made the TS increase from 8.2 to 14.7 MPa, but the film elongation decrease from 30% to 6% [85]. Coupling thermal treatment with irradiation could make the mechanical properties of soy protein films improve significantly [83].

In general, the hydrophilicity of proteins and plasticizers make the soy protein-based films show poor water vapor barrier properties, good oxygen barrier properties and moderate mechanical properties [69,85].

### 2.3. Zein

Zein, that is a by-product during corn processing, accounts for about 45–50% of corn proteins [86,87]. Zein proteins are soluble in alcohol, but not in water because of the existence of nonpolar amino acids in zein, which contributes to the water vapor barrier properties of zein films [86,88,89,90,91]. However, the films made from pure zein are brittle, yet various measurements proposed by researchers can be carried out to improve the properties of zein films [92,93].

“Wet or solvent process” can be applied to prepare zein-based films, and polyols or fatty acids are commonly chosen as the plasticizers. First, an appropriate solvent is chosen for dissolving zein, plasticizers and some other agents. Then, the film-forming solution is cast on a surface that is flat and nonstick. Finally, the solution is evaporated and a free-standing film can be peeled off from the surface [94]. The zein films can also be prepared through the combination of ”wet” and ”dry”, e.g., stretching or extruding the moldable resin prepared from oleic acid into a film [95,96].

The film-forming solutions for zein include water, methanol, ethanol and acetone, but only water, ethanol and the solution combination of the two, can be used for food packaging [97]. Ethanol hydrous solution is usually chosen as the film-forming solution, and the properties of zein films indicated that the films prepared in acetone were stronger but less flexible than those prepared in ethanol [98].

Physical treatments such as UV irradiation and **γ**-radiation can be employed during or after zein films formation to cure the films. The film structures can be affected through the curing method and thus the film properties can be changed accordingly [94,99].

Commonly, zein-based films have a moderate moisture barrier, oxygen barrier and mechanical properties [69].

### 2.4. Casein

Casein, one of the milk proteins, contains four main subunits: kappa-casein, beta-casein, alpha s1-casein and alpha s2-casein that make up 13%, 36%, 38% and 10% of the casein composition, respectively [60,100]. The unique properties of the four protein fractions affect the film forming ability of casein [101].

Casein can easily form films from aqueous solutions without further processing because of the strong interchain cohesion caused by their random-coil nature and a great number of formed intermolecular hydrogen, hydrophobic and electrostatic bonds [102]. There are such properties of casein, such as biodegradability, high thermal stability, nontoxicity, the ability to bind small molecules and ions, micelle formation capability, all making the protein a good material for biodegradable films [103]. Due to the water solubility, emulsification capability and their high nutritional value, caseins are desirable biomaterials for the preparation of edible films [104,105,106,107]. Additionally, casein is readily available [108]. Compared to other proteins, caseins are more insoluble, which make caseinate (mainly sodium caseinate), and this is often used as an alternative to traditional packaging materials. Although casein-based films have mentioned above advantages as food packaging, some defects still need to be improved before they can be applied.

**Figure 4:**
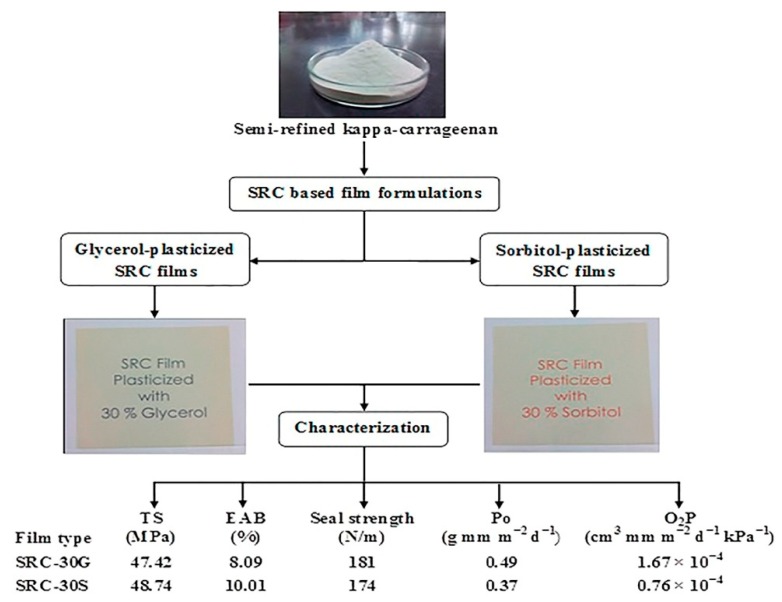
The addition of sorbitol or glycerol could reduce shrinkage during drying and improve the properties of edible films. Reproduced with permission from [109].

A kind of cohesive film matrix produced by the interactive forces between nonpolar and polar amino acid in the structure of casein will shrink during the drying process and then become brittle. The addition of edible plasticizers, e.g., sorbitol or glycerol, can solve the problem (as shown in Figure 4) [109]. However, the plasticizer concentration has a great effect on the tensile properties of films, and the tensile strength would increase with the decrease of concentration.

Polar amino acids are distributed along the casein chain, which makes the casein-based films have a good barrier effect upon non-polar molecules such as oxygen, and a good protective effect on foods that are prone to oxidation [21,106].

Most casein-based films are soluble in water and highly sensitive to moisture, which seriously affects their mechanical properties and barrier properties. Even though the casein-based films have been plasticized, they still cannot show good mechanical properties and elasticity. The modification of the polymer network through physical and chemical treatments can make the films functionality be improved [105,110]. Glutaraldehyde [111], transglutaminase [112,113], genipin [114,115,116], tannic acid [117] and wax [107] are typical chemical agents those are used as crosslinkers.

Besides, other methods for the improvements of films prepared from casein and its derivatives have been reported, such as combination with polysaccharides [118,119] or lipid [109,120,121], pH alteration [122], photo-induced polymerization [123], pulsed light [124], and so on.

### 2.5. Whey

Whey, another milk protein, is a by-product during cheese manufacturing, which makes the protein receive much attention. Whey-based films can be obtained from whey protein concentrates (WPC) and isolates (WPI). The protein contents of the two materials are different, respectively, at least 90% and 50–80%, and both of the two are rich in cysteine, methionine and sulfur-containing amino acids [125].

The film-forming of whey-based films depends on the thermal denaturation of whey proteins in aqueous solutions. The three-dimensional structure of whey protein can be modified through heating, making the internal hydrophobic and SH groups be exposed which have the hydrophobic and intermolecular S–S bonding interactions promoted upon drying [126,127]. The plasticized WPI films can be obtained through heating the whey protein solution with a concentration of 8–12% at 75–100 °C for a few minutes [128]. In this way, the irreversible denaturation of whey proteins takes place and the formed films have consistent structure [129]. As long as the amount of solids deposited in each casting surface unit can be kept constant, good whey-based films can be prepared from the WPI solution with a lower concentration (down to 5% (*w*/*w*)) [130]. 

Adjusting the denaturing temperature and pH of the film-forming solution could make the film-forming conditions of WPCs optimized; e.g., when the pH of film-forming solutions was adjusted to 6.6 through 2 M NaOH produced solutions, the heating temperature and heating time were controlled at 75 °C and 30 min, respectively, to ensure uniform film [131].

Some other physical and chemical methods can also improve the properties of the whey-based films; for example ultraviolet (UV) radiation, alkalization and ultrasounds (US). UV treatment applied to film-forming solution at high dose makes most mechanical properties of the whey-based films significantly improved; i.e., the tensile strength, puncture deformation, elastic modulus and puncture strength increased. UV radiation acting directly on the whey-based films makes the color of the films become more yellow, greener and darker, compared to the untreated films. When UV radiation is applied to the film-forming solution, the effects on color are higher [132]. The pH could influence the properties of the whey proteins and whey-based films. Alkaline pH promotes protein denaturation, solubilization and unfolding [133]. Whey-based films can be prepared through making the unheated WPI film-forming solutions exposed to strongly alkaline conditions. Compared with the films prepared through heating the WPI film-forming solutions, the films obtained in the strongly alkaline conditions have lower physical strength and slightly weak water barrier properties, but are more likely to disperse in food cooking [134]. When both UV radiation and alkalization are applied in WPC film-forming solutions, in the case wherein the pH of the film-forming solution is not very high, e.g., 7 or 9, UV radiation makes the films become so strong that the puncture resistance of the films is improved; but in the case of the pH of film-forming solution being higher, e.g., 11, UV radiation cannot make the mechanical properties of the films improved, because high alkalization in the film-forming solution has made the degree of aggregation and denaturation so intense [135]. US treatments can also make the whey-based films become strong, and the more exposure time, the stronger the films [136].

The whey-based films made with plasticizers are flexible, transparent and bland, and have excellent barrier characteristics for oxygen, oil and aroma. However, the hydrophilic character makes the whey-based films poor moisture barriers. The addition of lipid materials (fat and oils) could increase the hydrophobicity of whey-based films and make the poor moisture barriers improved. The lipid materials commonly used include plant oils [137,138,139], waxes [140,141,142], fatty acids [143] and acetylated monoglycerides [144].

### 2.6. Gelatin

The partial degradation of collagen can produce gelatin, and the differences in degree of hydrolysis leads to different molecular weights of gelatin, basically between 65,000 and 300,000 g/mol. The main ingredients of gelatin include proline, 4-hydroxyproline and glycine, but different sources cause different contents of these ingredients; for gelatin from pigskin, for example, the contents are 13%, 9% and 33%, respectively [145]. The good film-forming properties, wide existence, low cost, biocompatibility and biodegradability make gelatin become an important raw material for the preparation of biodegradable films [146].

**Figure 5:**
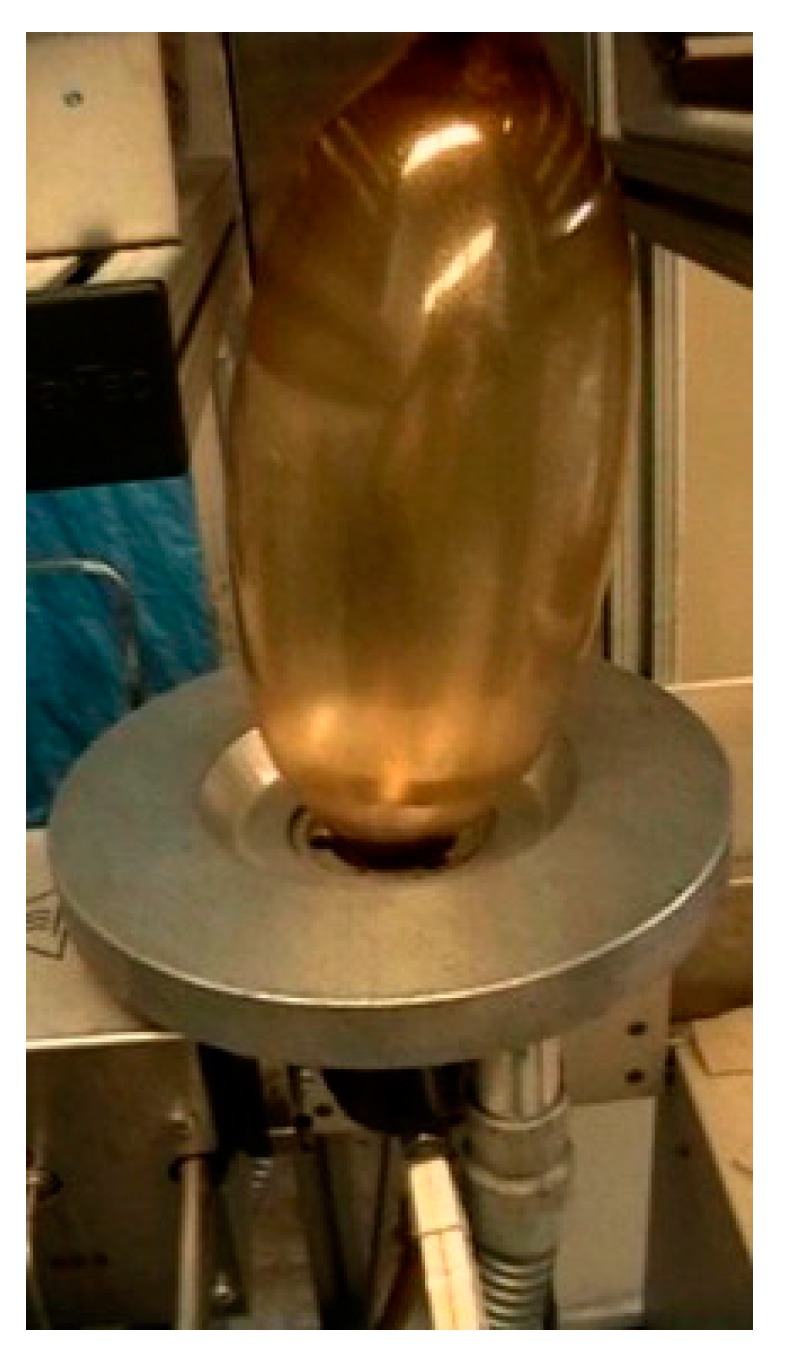
Gelatin films fabricated by blown-extrusion using yucca extract and glycerol. Reproduced with permission from [149].

Gelatin films can be obtained through casting from the gelatin aqueous solution. According to the different preparation temperature, it can be divided into cold-cast films and hot-casting films, where the former is prepared under the condition of less than or equal to room temperature, and the latter is prepared at more than 35 °C. The conformational state of the two kinds of gelatin film is different; the former has a spiral structure, and the latter has a statistical coil structure. The latter films are more brittle than the former [147,148]. he gelatin films also can be obtained by extrusion and blown-extrusion (as shown in Figure 5). The thickness of gelatin films prepared by the above three methods is different, ranging from 357 to 55 μm, and the thickness of the films obtained by casting are thinner. The tensile strength of films prepared by casting is higher, while the extensibility of films prepared by extrusion is stronger [149].

Gelatin-based films are transparent, oxygen impermeable and the rmoreversible. The melting point of gelatin is close to body temperature, making the gelatin-based films become particularly important raw materials for the preparation of edible films [11]. Edible films based on gelatin have relatively low oxygen permeability and may possess antioxidant and antimicrobial properties by adding some agents like citrus essential oils, carvacrol, and so on [150,151,152].

## 3. Protein-Based Biopolymers Applications for Foods-Packaging

The choice of food packaging materials depends on the characteristics of packaged food. Different types of foods with different characteristics require different requirements for packaging materials. Besides, the choice of food packaging materials must take environmental factors into consideration, such as relative humidity, temperature and light intensity, to which the foods are exposed during distribution and storage [3].

### 3.1. Fruits and Vegetables

Vegetables and fruits are composed of living tissues that continue to undergo physiological and biochemical changes after harvesting, adversely affecting their quality and shelf life. Respiration and transpiration are the main factors affecting the shelf life of vegetables and fruits. Oxygen is necessary for respiration to take place. Once respiration takes place, the carbohydrate content and weight of vegetables and fruits will decrease, affecting the quality and taste of products [153,154]. Water loss by transpiration is the main cause of products’ deterioration, making vegetables and fruits lose weight, nutritional value and undergo an appearance change, such as turgidity, wilting, color and texture [15]. Shelf life can be regulated by controlling the respiration and transpiration rates of fresh fruits and vegetables; the lower the rate, the longer the shelf life [155].

In order to have the shelf life of vegetables and fruits extended, the rate of respiration and transpiration must be reduced through controlling relative humidity, temperature, light, surrounding gas (O_2_, CO_2_ and ethylene (C_2_H_2_)), and so on. Some of these factors can be controlled by appropriate food packaging, such as relative humidity, O_2_ and CO_2_ concentration. 

Different kinds of vegetables and fruits require different relative humidity. For most fruits and vegetables, the optimal relative humidities are 85–95% and 90–98%, respectively, and the ideal relative humidity for some root vegetables is up to 100% [156]. The relative humidity can be adjusted by the food packaging. Commonly, a high moisture barrier property causes a high relative humidity in the package [157,158].

The oxygen concentration reduced to less than 10% can make the respiration rate of vegetables and fruits controlled. When the oxygen concentration is excessively low, the anaerobic respiration occurs, the flavors and odors disappear and the anaerobic bacteria grow rapidly. Low concentration of O_2_, high concentration of CO_2_, or both, may reduce the production of C_2_H_2_ [159]. 

However, just regulating the concentration of carbon dioxide does not necessarily lead to an ideal ethylene concentration [160]. Therefore, the gas composition in the food packaging must be strictly controlled. The atmosphere conditions for the preservation of fresh fruits and vegetables are recommended in Table 3.

The protein-based films and coatings were widely used in the preserving of fruits and vegetables. The application of zein coatings to tomatoes makes the color change, softening and weight loss delayed without ethanol production [162]. Applied to apples and pears, the effect of zein coatings on the respiration rate is different, where the respiration rate of the former is decreased, but the respiration rate of the latter is increased. However, the weight losses of the two kinds of fruits are both delayed [163]. The fat deterioration of peanuts can be prevented through coating with a composite of gluten and SPI [164]. Gelatin-based films and coatings are often used to prolong the shelf-life of some kinds of vegetables and fruits, such as carrots [165], calyx from physali [166], cherry tomatoes [167], peppers [168], banana and eggplant [169], fresh-cut melons [170], strawberries [171], pineapple fruit [172], blueberry fruit [173] and minimally processed persimmon [174].

### 3.2. Dairy Products

Dairy products include milk, fermented milk products, processed cheese and cream, being those that provide the human body with numerous nutrients by an easy and quick way in our diet. Due to the perishability, the influence of external conditions such as oxygen, light, microorganisms and moisture cannot be ignored.

Oxidation and microbial growth in dairy products can be caused by high oxygen concentration. Nutrient loss, discoloration and off-flavor formation can be brought out by light-induced oxidation, and the presence of moisture accelerates the deterioration of dairy products. These require that the packaging materials of dairy products have low oxygen, moisture permeability and light resistance.

Cheese is one of the most studied and challenging dairy products due to its diversity and differences in characteristics. The instability caused by its biochemically and biologically dynamic nature [175] makes the edible films and coatings widely applied to prolong the shelf life of kinds of cheese.

The selection of proper packaging materials for cheese is challenging, because there are numerous biochemical and microbial processes involved in cheese production, such as lipolysis, oxidation, proteolysis, weight loss and moisture content, which affect flavor, texture and all other physical and chemical properties [176,177].

The barrier properties of packaging materials have a great impact on the shelf life of cheese. Cheese packaging requires gas exchange, because cheese consumes oxygen and releases CO_2_, but packaging materials must have low oxygen permeability to prevent the growth of harmful microorganisms and the oxidation of fats. Packaging materials should be properly permeated with carbon dioxide, because the presence of carbon dioxide will prolong the growth lag of spoilage microorganisms. Some studies on carbon dioxide permeability show that high and low permeability have different advantages and disadvantages [152]. Cheese should also avoid water evaporation and light-induced oxidation, in order to extend the shelf life of cheese, and avoid the deterioration caused by the oxidation of fat [153,154]. Table 4 shows the applications of the protein-based films and coatings for the packaging of dairy products and their potential impact on the quality and shelf life of various cheeses during storage.

### 3.3. Meat and Products

Meat, including fresh meat and cured meat products, is one of the sources of animal protein for most people in the world. Proper packaging allows meat to maintain its protein quality. 

For fresh meat, O_2_ and CO_2_ concentrations must be in the suitable range. The red oxymyoglobin color can be ensured under high O_2_ concentration on the product surface, and that can be achieved by using high oxygen permeability packaging materials or Modified Atmosphere Packaging (MAP) with high concentration of O_2_. However, high levels of oxygen make Gram negative bacteria develop fast on the surface of fresh meat, especially for the ground meats, which have a considerable surface area. The traditional microbial growth can be inhabited under High O_2_ (70–80%) coupled with high CO_2_ (20–30%) in MAP, but the lactic acid bacteria, e.g., *Carnobacteria* spp., can grow slowly in such atmosphere [3]. For some meats with lighter color, the longer shelf life can be achieved by using higher CO_2_. However, some researches show that off-flavor and discoloration may be formed in poultry when the CO_2_ concentration is more than 25% [184].

For cured meat products, the most important problem in storage is to prevent deterioration caused by discoloration. In the presence of light and oxygen, the cured meat products may fade within hours [185,186]. The increase of partial pressure of O_2_ directly causes the acceleration in photo oxidation of the pigment [187]. The reduction in color deterioration may be achieved by the vacuum packaging or MAP under anoxic atmosphere. When there is a very low concentration of residual O_2_, the packaged cured meat products can be left in the dark for at least four days, the respiration of microorganisms and tissues can consume the residual O_2_, and then the packaged cured meat products are placed in the display light, which can effectively reduce the discoloration [188,189].

In order to ensure the safety and quality of the meats and products, the biopolymer packaging, which is environmentally friendly, is widely used, and some packaging methods were developed, e.g., vacuum, aseptic, modified atmosphere, intelligent, active, and so on. Some application examples of protein-based films for meat and products are shown in Table 5.

### 3.4. Frozen Foods

The deterioration reactions of frozen foods during storage are mainly chemical, including the degradation of vitamins and pigments, destabilization of proteins and oxidation of lipids. Proper packaging can effectively prevent the deterioration of frozen food. Packaging that protects frozen foods from light and oxygen has been shown to reduce the oxidation of pigments and lipids in frozen salmon ides [199] and frozen prawns [200]. When the enzymes have not been removed by blanching (for example, from vegetables), enzyme-catalyzed oxidation may occur without an effective oxygen barrier. Moisture loss caused by the sublimation of the surface of the frozen foods leads to the impairment of visual appearance, unacceptable weight losses of frozen foods and freezer burns. Sublimation of frozen foods can be avoided by sticking packaging materials that are highly impermeable to water vapor tightly to the surface of frozen foods. Obviously, the packaging materials need to be able to withstand low temperatures for a long time. Additionally, the packaging materials should possess grease barrier properties when they are applied in frozen foods with high fat contents [3].

Frozen fish is an important frozen food. Freezing is a good way for fish preservation that can prolong the shelf life of fish with minor changes in product quality if necessary precautions are taken. The freezing process generally has a significant impact on the quality of the final product. During the freezing process, some undesirable modifications may occur, such as lipid oxidation, weight loss, protein denaturation and freeze burning [201,202]. The application of appropriate packaging can prevent some of the undesirable changes.

The whey protein-based coatings applied on Atlantic salmon fillets after freezing make the thaw yield increase, the drip loss decrease and the color parameters change, in comparison with those applied before freezing. The lipid oxidation of salmon fillets is also delayed with the application of protein coatings [203]. The lipid oxidation of salmon fillets can be significantly delayed through the application of ultrasound treatment to the whey protein-based coatings, compared to the samples coated with untreated whey proteins and the uncoated samples [202]. The addition of microbial transglutaminase (MTG) into the heated whey protein-based coatings is also effective for delaying lipid oxidation [204]. 

The application of whey protein-based coatings with sodium alginate to kilka fish can enhance the product quality and prolong the shelf life during frozen storage up to 6 months. Total bacteria count and *Staphylococcus* bacteria counts (2/51 and 1/44 log CFU/g) become less than those in control samples (3.21–2.28 log CFU/g). Free fatty acids, peroxide value, thiobarbituric reactive substances (TBRS), pH and total volatile nitrogen (TVN) in coated samples significantly decrease compared with the control (*p* < 0.05) [205].

In addition, the excellent hydrophilicity of proteins allows the proteins-based films to adhere well to the surface of frozen foods and provides a barrier to the diffusion of oxygen and carbon dioxide without stopping the diffusion of water [203,206,207].

## 4. Protein–Based Active Materials

With the increasing consumer demand for packaged food that is mild, fresh, tasty, convenient and has a long shelf life, the development of new packaging systems is increasingly urgent. In addition, the globalization of markets has led to longer distribution distances, which has created new challenges for the food packaging industry, i.e., longer shelf life and safety and quality issues in transportation.

Active packaging plays an increasingly important role in the food packaging industry due to its unique function. The function of active packaging occurs during the interaction between the product package and the product environment, which not only improves the safety and/or sensory attributes of the product, but also prolongs the shelf life of the products [208,209,210]. Active packaging refers to the addition of antibacterial and antioxidants agents to food packaging, so that food packaging has antibacterial and antioxidant properties, and the shelf life of food packaged with this packaging can be prolonged [211]. The amphiphilic nature of protein-based films allows it to act as carriers for active compounds to preserve the quality of packaged foods [212]. Additionally, the control of the release of active and volatile mass transfer across the film determines the active effect or food quality. Therefore, it is very important to understand the release mechanism of active compounds for the preparation and application of active films.

### 4.1. Release Models Applied to Active Packaging

The affinity, sorption and diffusivity of the protein-based films matrix determine the degree of protection of the active packaging for food quality. Affinity is the result of physicochemical interaction and is the main factor controlling mass transfer. Adsorption is the affinity of different compounds to polymers. Diffusion describes the molecular motion of compounds in the polymer network, depending on a number of factors, such as the structural characteristics or molecular weight of the matrix. The adsorption mechanism involves the migration of small, volatile molecules without interactions, while the diffusion mechanism involves the migration of molecules with large molar volumes. Generally, the low affinity of volatile compounds and the low diffusivity of protein-based films bring out a higher mass transfer efficiency [213,214].

The kinetic studies on the release of active compounds in the films indicate that kinetic data can reveal the ability to release the active compounds to the protein-based films. Therefore, the diffusivity could be obtained from the release kinetics based on Fick′s Second Law [213,215]:

(1) $$\frac{\partial C}{\partial t}=D_{P}\frac{\partial^{2}C}{\partial x^{2}}$$

where *C* is the concentration of the active compounds in the film, *t* is the time and *D_P_* is the diffusion coefficient of the active compounds across the film. Through comprehensive consideration and certain model simplification the amount of a substance migrating from a polymer film into food/simulated food could be obtained based on Equation (2) [216]:

(2) $$M_{F,t}=M_{F,\infty }\left\{1-\sum_{n=1}^{\infty }\frac{2\alpha (1+\alpha )}{1+\alpha +\alpha^{2}q_{n}^{}{}^{2}}\exp\left[\frac{-q_{n}{}^{2}D_{P}t}{L_{P}{}^{2}}\right]\right\}$$

where *M_F,t_* is the amount of active compounds released into food/simulated food at time *t*; *M_F,_*_∞_ is the amount of active compounds released into food/simulated food at equilibrium; α = *V_F_*/(*V_P_·K_P,F_*) and *L_P_* is one half the thickness of the film, *V_F_* is the volume of food/simulated food, *V_P_* is the volume of the films; *K_P_*_,*F*_ = *C_P_*/*C_F_* is the partition coefficient, *C_P_* and *C_F_* are the concentration of the substance in films and in food/food stimulants at equilibrium, respectively; and *q_n_* are the non-zero positive roots of tan (*q_n_*) = −*q_n_*·α. However, at the end of the experiment, when the release speed is slow and equilibrium is not reached, Equation (3) can be adopted under the boundary condition of *M_t_*/*M_p_* < 0.6 [217]:

(3) $$\frac{M_{t}}{M_{P}}=\frac{4}{L_{P}}{\left(\frac{Dt}{\pi }\right)}^{1/2}$$

where *M_t_* and *M_p_* are the mass and initial loading of active substance in the film, *D* is the diffusion coefficient and the value is estimated according to the slope of the plot of *M_t_*/*M_p_* versus *t*^1/2^.

The release rate of active compounds is closely related to temperature. The higher the temperature, the faster the release rate. The diffusion coefficient of the active compounds at a certain temperature can be obtained by Equation (4):

(4) $$D_{P}=D_{0}\exp\left(-\frac{E_{D}}{RT}\right)$$

where *E_D_* is the activation energy of diffusion, (J/mol); *R* is the gas constant, its value is 8.314 J/mol/K; *T* is the temperature, K; and *D*_0_ is the pre-exponential factor, cm^2^/s. The activation energies could be obtained through the Arrhenius plot [215,218]. After the activation energy is obtained, the diffusion rate of the active compounds could be estimated. The lower activation energy, the more rapid release of active compounds from films [215].

### 4.2. Antimicrobial Protein-Based Films

Adding antimicrobial agents to biopolymer films can create an environment inside the packaging that inhibits or delays the growth of microorganisms on the surface of a product, and thus extends the shelf life of the product. By using antibacterial films, the product’s surface can maintain a high concentration of active compounds. There are volatile and non-volatile antimicrobial agents, the former evaporating in the package and floating to the headspace of the package, and the latter diffusing into the product. The compatibility and thermal stability of materials should be considered in the selection of antimicrobial agents. There are many kinds of antibacterial agents applied in food packaging, and most of them come from natural resources.

The substances obtained from natural resources that can be used in protein-based films as antimicrobial agents include bacteriocins, Ethylene Diamine Tetraacetic Acid (EDTA), acidulant, antimicrobial enzymes, plant extracts, Essential Oils (EO), Metallic Nano Particles (NP) and so on [219]. There are various antimicrobial mechanisms of EO for affecting microbial cells, including disrupting enzyme systems, attacking the phospholipid bilayer, and destroying genetic material of bacteria [220]. NPs have many advantages as antimicrobial agents, such as stability, low volatility and the broad spectrum against foodborne pathogens [221,222]. The concentration of NPs used as antimicrobial agents is so low, that the toxicity toward human cells can be negligible [223].

The addition of antibacterial agents not only makes the protein-based films antibacterial, but also affects the performance of the films. Table 6 shows the application examples of some antibacterial agents.

### 4.3. Antioxidant Protein-Based Films

The addition of antioxidants to packaging materials can delay the oxidation of lipids and reduce odors, discoloration and vitamin loss of food. Besides, compared with adding antioxidants directly to food, the technique has several advantages, such as less antioxidant dose and antioxidant activity concentrating on more sensitive product surfaces by migration from the film to the food.

The use of this antioxidant film extends the shelf life of the product and omits additional processing steps such as mixing, soaking or spraying [209,247].

Antioxidants used in food packaging are either natural antioxidants or derived from natural resources. Synthetic antioxidants have been banned from food packaging because of their safety concerns. Similar to the antibacterial protein-based films, the addition of antioxidants not only changes the antioxidant property of the protein-based films, but also changes some performance of the protein-based films.

Ascorbic acid [232], α- tocopherol [234,248,249], coumarin [250] and ferulic acid [251] are natural antioxidants. Among them, α- tocopherol has the greatest influence on the properties of the protein-based films. The steric effect of α-tocopherol is so great, that the diffusion of gases through the protein chains is limited. In other words, the mechanical and barrier properties of the protein-based film can also be improved by the addition of antioxidant compounds.

Besides, there are some natural extracts that act as antioxidants, which are an important part of antioxidants obtained from natural resources. The selection of natural extracts depends on the characteristics of the packaged product and the requirements of storage. Myofibrillar-protein films coupled with Catechin-Kradon leaf extracts could effectively make the lipid oxidation and the formation of the metmyoglobin of refrigerated fish slices suppressed [251]. In addition to improving the antioxidant property of the films, natural extracts could have a certain effect on the film performance. The addition of Red-grape extracts into soy-protein films made the flexibility and the water vapor permeability of the casted films to be improved. The reason was that the redistribution of hydrogen interactions was induced and the interactions of protein–protein were replaced by those of protein–polyphenol, which are particularly noticeable when compression molding is applied. At the same time, because of the strong interactions between the phenolic compounds and the protein matrix, more antioxidants were released from the compression-molded films [252]. The addition of *Caesalpiniaspinosa* and *Caesalpiniadecapetala* extracts into gelatin films would decrease strength and increase flexibility [253]. The incorporation of green tee extracts into gelatin films made the flexibility, water vapor permeability and water solubility decrease and the strength increase [254]. However, when black-tea, green-tea and oolong-tea extracts as antioxidants were incorporated into protein-based films, themselves produced from distiller dried grains for preventing the lipid oxidation of pork meat during storage, the flexibility and the strength of the films were changed, the former increased and the latter decreased [255]. Adding mango seed-kernel extracts into the protein-based films produced from SPI and fish gelatin makes the antioxidant activity and strength increase, while the flexibility and water solubility decrease [20,247]. The addition of murta-leaf extracts into gelatin films also changed the properties of the films, and the higher the polyphenol content, the more the mechanical properties decreased [256].

EOs are bioactive compounds formed by a mixture of various substances, with a wide range of chemical components, obtained from plant secondary metabolism. In addition to their traditional roles as natural food additives and aromatic agents, they can also be used as antibacterial agents and antioxidants [257,258]. The typical antioxidants in EOs include clove [242,259], marjoram and coriander [242], Zataria multiflora Boiss [260], ginger [261], *Morinda citrifolia* [262], orange leaves [237], cinnamon [238,239], garlic [263], and so on. There are also many studies and applications of adding EOs to protein-based films for delaying the oxidation of food. The delay of lipid oxidation of sliced cheddar cheese can be achieved by adding coriander, marjoram or clove EOs to gelatin-based films [242]. The shelf life of fresh shrimp can be prolonged by covering gelatin-based films incorporated with orange leaf EOs [238], that of fish fillet can be prolonged by covering WPC coating incorporated with cinnamon EOs [239], and that of smoked salmon during refrigerated storage can be prolonged by covering composite protein-based films incorporated with clove EOs [259].

## 5. Conclusions

The good film-forming properties of proteins make the protein-based films become one of the key points in the research of food packaging materials. Due to different physical and chemical properties, the film-forming ability of each protein is slightly different. The preparation methods of various protein-based films are similar. The corresponding plasticizers are usually added to improve the elasticity and viscoelasticity of protein-based films in the production process. In general, the protein-based films have moderate mechanical properties and good oxygen barrier properties, but they are sensitive to water. Some physical or chemical post-treatment methods can be applied to make the properties of protein-based films improved for specific applications.

Each type of food has specific storage requirements, which requires packaging materials to meet specific storage conditions for specific foods. Protein-based biopolymer materials can meet the requirements of various food packaging materials through modification. Additionally, the nutrition and biodegrade ability of proteins makes themselves become one of the best choices of food packaging materials. The research about the relations among the function, structure and composition of protein-based films will guide the methods and schemes of film modification, so that the protein-based films suitable for food packaging with better properties will be prepared.

Active packaging provides a guarantee for the rapid development of the functional food market. The active (antioxidant and antimicrobial) protein-based films, those selected based on the food characteristics and storage requirements, can effectively inhibit or delay the growth of microorganisms and the oxidation of lipids, thus ensuring food safety and prolonging the shelf life of food.

## Figures and Tables

**Table 1:** Natural plasticizers used in protein-based films and coatings.

System of Application	Plasticizer	References
Zein	Oleic and linoleic acids	[47]
Whey protein	GLY and sorbitol	[51]
Wheat gluten	saturated fatty acids	[45]
	Glycerin	[52]
Caseinate-pullulan	Water and sorbitol	[34]
Whey protein/beeswax emulsion	GLY	[53]
Gelatin	GLY and sorbitol	[53]
	Sucrose, oleic acid, citric acid, tartaric acid, malic acid, PEG of different molecular weights (300, 400, 600, 800, 1500, 4000,10,000 and 20,000), sorbitol, mannitol, EG, DEG, TEG, EA, di ethanol amine (DEA) and TEA	[35]
Pigskin gelatin	GLY	[54]
	Sorbitol	[55]
Bovine gelatin	Fatty acids	[56]
	Sorbitol	[55]
	GLY	[57]

**Table 2:** Biopolymer and common commercial films, properties reported.

Films	Plasticizers	Opacity (A.nm)	Mechanical Properties (TS in MPa)	Thermal Properties	Water Vaper Permeability	References
Wheat gluten						
Gliadins	Gly 35%	~34	%E = ~390 TS = ~7	NR	~7 × 10^11^ [(gm)/(m^2^ s Pa)]	[58]
Glutenins	Gly 35%	~101	%E = ~250 TS = ~1	NR	~4 × 10^11^ [(g m)/(m^2^ s Pa)]	
Other Sources						
Zein	Gly 40%	NR	%E = ~118 TS = ~4	*T*_g_ = ~30 °C	~4 (g mm/m^2^ h kPa)	[59]
Kafirin	Gly 40%	NR	%E = ~24 TS = ~1	*T*_g_ = ~30 °C	~8 (g mm/m^2^ h kPa)	
Avenin	Gly 40%	NR	%E = ~40 TS = ~4	*T*_g_ = ~28 °C	~3 (g mm/m^2^ h kPa)	
Milk						
Casein	Gly 50%	NR	%E = ~65 TS = ~2.5	NR	~7 (g mm/m2 h kPa)	[60]
Whey fraction						
WPI	Gly 40%	NR	%E = ~33 TS = ~0.9	*T*_g_ = ~50 °C	~8 (g mm/m^2^ d kPa)	[61]
WPC	Gly 40%	NR	%E = ~18 TS = ~0.7	T_g_ = ~43 °C	~10 (g mm/m^2^ d kPa)	
Synthetic polymers						
High Density Polyethylene (HDPE)	NR	NR	%E = ~600 TS = ~54	T_g_ = ~80 °C	~6 (g/m^2^ d)	[21]
Low Density Polyethylene (LDPE)	NR	NR	%E = ~300 TS = ~27	T_g_ = ~−125 °C	~18 (g/m^2^ d)	
Polypropylene (PP)	NR	NR	%E = ~150 TS = ~151	T_g_ = ~−10 °C	~8 (g/m^2^ d)	
Polyethylene Terephthalate (PET)	NR	NR	%E = 70 TS = 79	T_g_ = ~76 °C	~21 (g/m^2^ d)	

**Table 3:** Recommended atmosphere conditions for the preservation of fresh fruits and vegetables [161].

Group	Commodity	CO_2_ (%) ^a^	O_2_ (%) ^a^
1	Potatoes	0	0
	Carrots	0	0
	Beets	0	0
2	Tomatoes	0	3–5
	Peppers	0	3–5
	Cucumbers	0	3–5
	Lettuce	0	2–5
	Celery	0	2–4
	Onions(dry)	0	1–2
3	Pears	0–5	1–3
	Lemons	0–5	5
	Apples	1–5	2–3
	Cauliflowers	2–5	2–5
	Artichokes	3–5	2–3
	Peaches	5	1–2
4	Others	5–15	1–5

^a^ Percentages are volume or mole percentage; the remainder is nitrogen.

**Table 4:** Applications of protein-based films and coatings for dairy products.

Product, Storage	Film	Added Values	Effects		References
Fresh *Kashar cheese*, 4 °C, 8 weeks	Zein (Z)/carnauba wax (5%) composite films (ZW)	Lysozyme (0.7 mg/cm^2^) (L)	*L. monocytogenes*	C and F-Z did not change significantly counts in the first 28 days, but the counts of these controls increased between the 28th and 56th days All samples containing lysozyme showed significant reduction No significant increase occurred in counts of cheese samples AF-Z-L, AF-ZW-L, AF-Z-MIX, AF-ZW-MIX	[178]
		Mixture of lysozyme (0.7 mg/cm^2^), catechin (3 mg/cm^2^) and gallic acid (3.0 mg/cm^2^) (MIX)	Lipid oxidation/TBARS	C > F-Z = AF-Z-L = AF-ZW-L (no significant effect) >AF-Z-MIX = AF-ZW-MIX (significantly lower)	
Unripened, creamy *Ricotta* cheese, MAP (40% CO_2_, 60% N_2_) at 4 °C, 30days	Chitosan/whey protein coating		pH	C = ACO (decrease, after 7, and remained relatively constant until 30 days)	[179]
			Titratable acidity	C (increased) > ACO (no significant differences)	
			LAB	C > ACO	
			Mesophilic acrobic bacteria	C > ACO	
			Psychrotrophic bacteria	C > ACO	
			Acidity	Delayed development by ACO	
			Sensory quality	No effect of ACO	
			Shelf-life	C < ACO	
Cheddar cheese, 5 ± 1 °C, 30 days	Casein (CS) Whey protein concentrate (WPC) films		Soluble nitrogen	C = F-CS = F-WPC (125.7–151.2 mgN_2_/100 g)	[180]
			TBARS	C (0.01–0.05) > F-CS = F-WPC (0.01–0.04)	
			Titratable acidity	C > F-CS = F-WPC	
			TVC	C = F-CS = F-WPC (7.8–8.1 log CFU/g)	
			Yeast, mold	C (1.1–1.9 log CFU/g) >F-CS = F-WPC (1.1–1.8 log CFU/g)	
			Sensory	No significant effect	
Semisoft, mini *RedBabybel*^®^ cheese, 4 °C, 1 week	Sodium caseinate film	Nisin (1000 IU/cm^2^ surface area AF)	*Inoculated product was put on active film for analyses *Listeria innocua*		[181]
			Surface-contaminated cheese	C > AF (1.1 log)	
			In-depth contaminated cheese, mm distance of film from contaminated spot	AF, 3 mm (0.25 log) > AF, 2 mm (0.9 log) > AF, 1 mm (1.1 log)	
*Cheddar* cheese, 65% RH, 10 °C, 5 days	Sodium caseinate (SC)		Psychrotrophic bacteria	C > F-SC = CO-SC > F-CH = CO-CH = F-SC/CH = CO-SC/CH	[182]
	Chitosan/sodium caseinate (SC/CH) films		Yeast	C > F-SC = CO-SC > F-CH = CO-CH = F-SC/CH = CO-SC/CH	
			Molds	C > F-SC = CO-SC > F-CH = CO-CH = F-SC/CH = CO-SC/CH	
Fresh *Kashar cheese*, 10 °C, 30 days	Wheat gluten (WG) methyl cellulose (MC) films	Natamycin 1.2 mg NA/10 g film solution 2.5 mg NA/10 g film solution 3.10 mg NA/10 g film solution 4.20 mg NA/10 g film solution	*A. niger*	C > F-MC (0.6 log) = AF-MC1 (no significant reduction) >AF-MC2 (2 log) = AF-MC3 = AF-MC4 C > F-WG (4.11 log) > AF-WG1 (completely inhibited) = AF-MC2 = AF-MC3 = AF-MC4	[183]

Abbreviations: ACO, Active coated sample; AF, active film; C, uncoated sample; CO, coated sample; F, film; log, log CFU/g reduction compared to control; MC, moisture content; TBARS, thiobarbituric acid reactive substances; TVC, total viable counts.

**Table 5:** Protein-based films and coatings for meat and products.

Product, Storage	Films/Coatings	Added Value	Effect		References
Fresh beef cuts: 5 °C, 12 days	Whey protein isolate	Cinnamon, cumin, thyme essential oil (TO)	TVC (shelf life)	C = F < AF-cinnamon (4–12 days) < AF-cumin (6–12 days) < AF with-thyme (8–>12 days)	[190]
Rainbow trout fillets vacuum: 4 °C, 26 days	Gelatin	LEO	TVC, psychrotrophic bacteria counts, *Enterobacteriaceae*, and LAB	C < F < AF 0.1% LEO < AF 1% LEO	[191]
			Color, pH increase, TVB-N, free fatty acid, PV, and TBARS	Preservative effect followed increasing order: C < F < AF 0.1% LEO < AF 1% LEO	
			Sensory shelf life	AF 1 % LEO (22 days) > AF 0.1% LEO = F (20 days) > C (15 days)	
Mackerel meat powder: 28–30 °C, 30% RH, 30 days	Gelatin with CNa lid sealed to aluminum cups	Coconut husk ethanol extract (CH)	Oxidation (PV, TBARS, and volatile compounds)	Decrease in AF–CNa–CH	[192]
			Moisture absorption	Decrease in AF–CNa–CH	
Ground beef patties vacuum: 4 °C, 12 days	Isolated soy protein	*Oreganum heracleoticum* (OR), *Thymus vulgaris* L. (TH) essential oil OR+TH ratio of 1:1	TBARS	No effect	[193,194]
			PV and free fatty acids	Lower values were determined for AF-OR or AF-TH particularly at later stages of storage	
			Color	Reduced, but acceptable, redness (a*) values	
			TVC, LAB, and *Staphylococcus* spp.	No effect of films	
			Coliform bacteria and *Pseudomonas* spp.	Reduced in AFs	
Fresh beef cuts: 5 °C, 12 days	Whey protein isolate	Sodium lactate (NaL); ε-polylysine (ε-PL)	TVC (shelf life)	C = F (6 days) <AF–*ε*–PL 0.25% = AF-NaL 1% (8–10 days) < AF-ɛ–PL 0.75% = AF-NaL 2% (10–12 days) V	[195]
			Pseudomonades counts	C = F > AF- ɛ–PL 0.25% = AF-NaL 1% > AF-ɛ–PL 0.75% = AF-NaL 2%	
			LAB counts	C = F = AF-NaL 1% = AF-NaL 2% > AF-ɛ–PL 0.25% > AF-ɛ–PL 0.75%	
Indian salmon fillets 6 °C, 16 days	Gelatin chitosan; T1: gelatin; T2: gelatin + chitosan + garlic extract; T3: gelatin + chitosan + lime juice	Lime extract; garlic extract	TBARS (shelf life)	C (8 days) < T2 < T1 = T3 (16 days)	[196]
			TVB-N (shelf life)	No effect of coatings (between 8 and 12 days)	
			pH increase	C = T1 > T3 > T2	
			TVC (shelf life)	C = T1 (8 days) < T3 (16 days) < T2 (above 16 days)	
			Psychrophilic count	C = T1 > T3 (2 log) > T2 (3 log)	
			Sensory shelf life	C (8–12 days) < T1 = T3 (12–16 days) < T2 (16 days)	
Rainbow Trout Fillets: 4 °C, 16 days	WPC	LPOS	TVB-N	Reduced	[197]
			Bacterial growth	Reduced	
			pH changes	Reduced	
			Lipid oxidation	No effect	
			Sensory shelf life	Extended by 4 days for ACO with 1.25% (*v*/*w*) LPOS; while 2.5%, 5%, and 7.5% LPOS ACOs showed moderate to high overall acceptability even until the 16th day of the storage period	
Grass carp fish balls: 4 °C, 20 days	Corn zein	Hexadentate 3-hydroxypyridinones (polymeric chelator)	Sensory properties	C < CO < ACO (similar till 10th day and than considerable differences)	[198]
			TVB-N	C > CO > ACO	
			TBARS	C > CO > ACO	
			TVC	C > CO (2 log) > ACO (4 log)	
			pH	ACO maintained stable pH during storage	
			Shelf life	C (7 days) < CO (13 days) < ACO (19 days)	

Abbreviations: ACO, Active coated sample; AF, active film; C, uncoated sample; CNa, cloisite Na+; CO, coated sample; F, film; LAB, lactic acid bacteria; LEO, laurel essential oil; LPOS, lacto per oxidase system; PV, peroxide value; TBARS, thio barbituric acid reactive substances; TVB-N, total volatile based nitrogen; TVC, total viable counts.

**Table 6:** Applications of antibacterial agents in protein-based films.

Antimicrobial Agents		Microorganisms	Performance Impact of Protein-Based Films	References
Bacteriocins	nisin	*Listeria monocytogenes*, *Pseudomonas aeruginosa*, *Yarrowialipolytica*, *Penicillium commune*, *Penicillium chrysogenum*	The strength was increased and the permeability was decreased.	[224,225,226,227]
	ε-polylysine	Spoilage flora of fresh beef	The strength was decreased and the flexibility was increased.	[228]
EDTA		*L. monocytogenes, Escherichia coli, Salmonella typhimurium, and Salmonella enteritidis*	There was a minimal effect on the mechanical properties.	[224,227,229]
Acidulant agents	sodium lactate, potassium sorbate, and citric, acetic, malic, lactic, tartaric, sorbic and paminobenzoicacids	*L. monocytogenes, E. coli, Salmonella gaminara, and Salmonella typhimurium*	The water-content equilibrium, water vapor permeability, and extensibility that affected the glass-transition temperature of the film were increased.	[230,231]
Antimicrobial enzymes	Lacto Per Oxidase System (LPOS) and lysozyme	*Shewanellaputrefaciens, Pseudomonas fluorescens, L. monocytogenes, Bacillus subtilis, E. coli, and Staphylococcus aureus*	The film structure and integrity were weakened, but when the concentration of active compounds was low, the film’s properties would not be affected.	[102,232,233,234]
EOs	lemon peel, Zataria multiflora Boiss, orange leaves, cinnamon, thyme, clove and oregano	Pathogens and food-spoilage microorganisms	The permeability, water solubility, strength and extensibility were decreased.	[235,236,237,238,239,240,241,242,243]
Commercially derived antimicrobials	ArticoateDLP-02, Artimex 152/NL, sodium octanoate, and Auranta FV	*E. coli, Bacillus cereus, P. fluorescens, S. aureus*, and microflora from beef steaks [32]	The protein network was destabilized.	[244]
	Ethyl-Nα-dodecanoyl-L-Arginate hydrochloride (LAE)	*L. monocytogenes* and *E. coli*	A barrier against carbon dioxide and oxygen was formed.	[218]
	Prunin Laurate ester (PL)	*L. monocytogenes, S. aureus*, and *B. cereus*	The functional properties were not affected.	[245]
NPs	Silver Nano Particles (AgNP)	foodborne pathogens	The barrier and mechanical properties were enhanced, but there might be potential toxicity.	[246]
